# Analysis of risk factors for peritoneal metastasis in patients with advanced gastric cancer

**DOI:** 10.1097/MD.0000000000049357

**Published:** 2026-06-26

**Authors:** Airu Su, Ran Guo, Yuhan Meng, Ye Li, Chao Wu

**Affiliations:** a Department of Oncology I, The Second Hospital of Hebei Medical University, Shijiazhuang City, Hebei Province, China; b Department of General Surgery III, The Second Hospital of Hebei Medical University, Shijiazhuang City, Hebei Province, China; c School of Nursing, Hebei Medical University, Shijiazhuang City, Hebei Province, China.

**Keywords:** advanced gastric cancer, Lauren classification, logistic regression, lymphovascular invasion, peritoneal metastasis, risk factors

## Abstract

Peritoneal metastasis is a common and adverse pattern of disease spread in patients with advanced gastric cancer, yet its clinicopathological determinants are not fully clarified. This retrospective observational study included 154 patients with histopathologically confirmed advanced gastric cancer treated at a single tertiary center between January 2020 and December 2024. Patients were categorized into a peritoneal metastasis group (n = 38) and a non-peritoneal metastasis group (n = 116) according to predefined diagnostic criteria based on contrast-enhanced computed tomography, staging laparoscopy, cytology, and intraoperative findings. Demographic, clinical, pathological, imaging, and intraoperative variables were extracted from electronic medical records, and comparisons between groups were performed using *t* tests and χ^2^ tests. Univariate and multivariate logistic regression analyses were used to identify independent risk factors for peritoneal metastasis. Baseline characteristics, including age, sex, body mass index, and major comorbidities, were comparable between groups, whereas Eastern Cooperative Oncology Group performance status ≥ 2 was more frequent in the peritoneal metastasis group. In multivariate analysis, tumor size ≥ 5 cm (odds ratio [OR] = 3.49, 95% confidence interval [CI]: 1.63–7.50), diffuse Lauren type (OR = 3.32, 95% CI: 1.49–7.42), lymphovascular invasion (OR = 2.59, 95% CI: 1.23–5.45), and peritoneal thickening or nodules on computed tomography (OR = 4.95, 95% CI: 2.17–11.28) were independently associated with peritoneal metastasis. These findings indicate that combining routine histopathological features with standard imaging signs may improve preoperative risk stratification and help select candidates for staging laparoscopy and individualized treatment strategies in advanced gastric cancer.

## 1. Introduction

Gastric cancer remains a major contributor to cancer-related mortality worldwide, and peritoneal metastasis represents one of the most frequent and lethal patterns of disease spread in advanced stages. Population-based data indicate that a substantial proportion of patients develop synchronous or metachronous peritoneal metastases during the disease course, which is associated with poor survival despite systemic therapy.^[1,2]^ Recent prognostic studies focusing on gastric cancer with peritoneal carcinomatosis have further underscored the dismal outcomes and the need to refine risk stratification in order to identify patients who might benefit from intensified or regional treatment strategies.^[3]^ Accurate preoperative identification of patients at high risk for peritoneal metastasis is therefore a critical issue in the management of advanced gastric cancer. Staging laparoscopy with peritoneal cytology is considered the reference method for detecting occult peritoneal dissemination; however, it is invasive, resource-intensive, and not feasible for universal application in all patients. Accordingly, several recent studies have sought to define clinicopathological predictors and to develop risk models that can guide the selective use of staging laparoscopy.^[4,5]^ These investigations have suggested potential roles for tumor size, depth of invasion, nodal status, and serosal involvement, but substantial heterogeneity in study design and variable selection has limited the generalizability of their findings.

Tumor biology also appears to play a key role in determining the propensity for peritoneal spread. Diffuse-type tumors in the Lauren classification, often associated with poorly cohesive or signet-ring cells, are more prone to transmural infiltration, lymphatic permeation, and peritoneal dissemination than intestinal-type tumors.^[6,7]^ Conventional histopathological markers of aggressiveness, such as lymphovascular invasion and perineural invasion, have been repeatedly linked to recurrence and adverse survival, but their specific contribution to peritoneal spread in advanced disease is less well-defined. Understanding how these pathological features, together with tumor burden parameters, relate to peritoneal metastasis could provide a more nuanced framework for risk assessment. In parallel, there has been growing interest in the use of imaging-derived biomarkers to predict occult peritoneal metastasis. Conventional computed tomography (CT) signs – ascites, peritoneal thickening or nodularity, and omental caking – are highly specific but variably sensitive for macroscopic peritoneal disease. Building on these features, recent machine learning–based radiomics models using contrast-enhanced CT have shown promising performance in diagnosing occult peritoneal metastasis or omental involvement in advanced gastric cancer, indicating that CT carries rich quantitative information related to serosal and peritoneal spread.^[8,9]^ Nevertheless, many of these models require specialized software, complex feature extraction, and external validation, which may limit their immediate applicability in routine clinical practice.

Against this background, there remains a clinical need for pragmatic, data-driven tools that integrate routinely available clinicopathological variables with standard CT findings to estimate the risk of peritoneal metastasis in patients with advanced gastric cancer. The present study addresses this gap by systematically evaluating demographic, tumor-related, histopathological, and imaging characteristics in a cohort of patients with advanced gastric cancer, with the aim of identifying independent risk factors associated with peritoneal dissemination and providing an evidence-based basis for individualized staging and treatment planning.

## 2. Materials and methods

### 2.1. Study design

This study was approved by the Ethics Committee of The Second Hospital of Hebei Medical University. This retrospective observational study included patients with histopathologically confirmed advanced gastric cancer who received treatment at our institution between January 2020 and December 2024. Patients were stratified into a peritoneal metastasis group and a non-peritoneal metastasis group based on the presence or absence of peritoneal dissemination. The study was conducted in accordance with the principles of the Declaration of Helsinki and was approved by the institutional medical ethics committee. Written informed consent was obtained from all participants prior to the use of their clinical data for research purposes.

### 2.2. Inclusion and exclusion criteria

Patients were eligible for inclusion if they met the following criteria: histopathologically confirmed diagnosis of advanced gastric cancer; complete clinical, laboratory, imaging, and intraoperative records available for evaluation; and documented assessment of peritoneal involvement through imaging, endoscopic evaluation, staging laparoscopy, or intraoperative findings.

Patients were excluded based on the following conditions: prior history of other malignant tumors; receipt of neoadjuvant chemotherapy, radiotherapy, or targeted therapy before initial evaluation, which might interfere with the assessment of peritoneal metastatic status; incomplete or missing key clinical data; evidence of severe hepatic, renal, or hematologic dysfunction that could affect clinical interpretation; and concurrent infectious, autoimmune, or systemic inflammatory diseases that could bias laboratory parameters.

### 2.3. Diagnostic criteria for peritoneal metastasis

Peritoneal metastasis was diagnosed based on comprehensive clinical evaluation using 1 or more of the following criteria: radiologic evidence of peritoneal dissemination on contrast-enhanced computed tomography, including the presence of peritoneal nodules, omental caking, ascites with irregular peritoneal thickening, or diffuse peritoneal enhancement; positive findings on staging laparoscopy, characterized by visible peritoneal or omental implants, nodular lesions, or seeding on the peritoneal surfaces; (3) cytological confirmation of malignant cells in peritoneal lavage or ascitic fluid obtained through laparoscopic or ultrasound-guided procedures; and (4) intraoperative identification of metastatic peritoneal lesions during exploratory or therapeutic surgery. A diagnosis was established when any one of these criteria was fulfilled.

### 2.4. Data collection

Clinical data were obtained retrospectively from the electronic medical records of all eligible patients with histopathologically confirmed advanced gastric cancer who received treatment at our institution between January 2020 and December 2024. For each patient, baseline demographic information (age, sex, body mass index [BMI]), lifestyle factors (smoking and alcohol use, if available), and comorbidities (including hypertension, diabetes mellitus, and coronary heart disease) were extracted. Functional status at diagnosis was recorded using the Eastern Cooperative Oncology Group performance status (ECOG PS). Tumor-related and pathological variables were collected from endoscopic, surgical, and pathology reports, including primary tumor location (proximal, middle, or distal stomach), maximal tumor diameter, pathological T and N categories according to the TNM staging system, Lauren classification (intestinal, diffuse, or mixed/indeterminate), histological differentiation (well/moderate vs poor or signet-ring cell), and the presence or absence of lymphovascular invasion and perineural invasion. Radiologic data were obtained from preoperative contrast-enhanced CT scans performed as part of routine staging. Two experienced radiologists, who were blinded to the final peritoneal metastasis status, reviewed the CT images and recorded the presence of ascites, peritoneal thickening or nodular lesions, and omental caking. Intraoperative findings, including the presence of visible peritoneal or omental implants, were abstracted from operative notes in patients who underwent exploratory or therapeutic surgery. All data were entered into a standardized database by trained investigators and cross-checked by a second reviewer to minimize transcription errors. Cases with missing key variables required for the primary analysis were excluded according to the prespecified criteria.

### 2.5. Statistical analysis

All statistical analyses were performed using IBM SPSS Statistics, version 26.0 (IBM Corp., Armonk). Continuous variables (e.g., age, BMI, tumor size) were expressed as mean ± standard deviation and compared between the peritoneal metastasis and non-peritoneal metastasis groups using the independent-samples *t*-test. Categorical variables (e.g., sex, ECOG performance status, T and N categories, Lauren classification, differentiation grade, lymphovascular invasion, perineural invasion, and CT/imaging findings) were summarized as counts and percentages and compared using the χ^2^ test or Fisher’s exact test when appropriate. Univariate logistic regression analysis was first conducted to evaluate the association between each clinicopathological or imaging variable and the presence of peritoneal metastasis, and odds ratios (ORs) with 95% confidence intervals (CIs) were calculated. Variables that reached statistical significance in univariate analysis or were considered clinically relevant were subsequently entered into a multivariate logistic regression model to identify independent risk factors for peritoneal metastasis. In the multivariate model, regression coefficients (β), standard errors, Wald χ^2^ statistics, *P*-values, adjusted ORs, and corresponding 95% CIs were reported. All statistical tests were 2-sided, and a *P*-value < .05 was considered statistically significant. Prior to analysis, dataset completeness was verified for all included variables. Because patients with missing key clinicopathological, imaging, or outcome data were excluded during study selection, there were no missing data among variables included in the final regression analyses.

## 3. Results

### 3.1. Baseline demographic and clinical characteristics

A total of 154 patients with advanced gastric cancer were included, comprising 38 patients in the peritoneal metastasis group and 116 patients in the non-peritoneal metastasis group. There were no significant differences between the 2 groups in age, sex distribution, BMI, or major comorbidities, including hypertension, diabetes mellitus, and coronary heart disease (all *P* > .05). The mean age was 59.2 ± 8.6 years in the peritoneal metastasis group and 59.6 ± 9.2 years in the non-peritoneal metastasis group (*t* = 0.25, *P* = .804). BMI was also comparable between groups (22.0 ± 2.7 vs 22.4 ± 3.1 kg/m^2^, *t* = 0.88, *P* = .380). In contrast, the proportion of patients with an ECOG PS ≥ 2 was significantly higher in the peritoneal metastasis group than in the non-peritoneal metastasis group (47.4% vs 24.1%; χ^2^ = 7.37, *P* = .007), indicating poorer baseline functional status among patients with peritoneal dissemination (Table 1).

**Table 1:** Baseline demographic and clinical characteristics of patients with and without peritoneal metastasis.

Variable	Peritoneal metastasis group (n = 38)	Non-peritoneal metastasis group (n = 116)	Test statistic	*P*-value
Age, yr, mean ± SD	59.2 ± 8.6	59.6 ± 9.2	*t* = 0.25	.804
Male sex, n (%)	26 (68.4%)	76 (65.5%)	χ^2^ = 0.11	.743
BMI, kg/m^2^, mean ± SD	22.0 ± 2.7	22.4 ± 3.1	*t* = 0.88	.380
Hypertension, n (%)	14 (36.8%)	49 (42.2%)	χ^2^ = 0.35	.557
Diabetes mellitus, n (%)	9 (23.7%)	21 (18.1%)	χ^2^ = 0.57	.451
Coronary heart disease, n (%)	7 (18.4%)	13 (11.2%)	χ^2^ = 1.32	.251
ECOG PS ≥ 2, n (%)	18 (47.4%)	28 (24.1%)	χ^2^ = 7.37	.007

BMI = body mass index, ECOG PS = Eastern Cooperative Oncology Group performance status, SD = standard deviation.

### 3.2. Tumor-related, pathological, imaging, and intraoperative characteristics

Tumor location (proximal, middle, or distal stomach) did not differ significantly between the peritoneal metastasis and non-peritoneal metastasis groups (χ^2^ = 1.50, *P* = .472). In contrast, tumor size was greater in the peritoneal metastasis group than in the non-peritoneal metastasis group (6.7 ± 1.8 cm vs 4.7 ± 1.7 cm; *t* = 5.85, *P* < .001). The proportions of T4 lesions (78.9% vs 50.0%; χ^2^ = 9.79, *P* = .002) and advanced nodal stage N2–3 (73.7% vs 42.2%; χ^2^ = 11.32, *P* < .001) were also higher in patients with peritoneal metastasis. Regarding histology, the diffuse Lauren subtype was more frequent in the peritoneal metastasis group, whereas the intestinal subtype predominated in the non-peritoneal metastasis group (overall χ^2^ = 15.16, *P* < .001). Poor or signet-ring cell differentiation (78.9% vs 54.3%; χ^2^ = 7.26, *P* = .007), lymphovascular invasion (76.3% vs 45.7%; χ^2^ = 10.78, *P* = .001), and perineural invasion (63.2% vs 38.8%; χ^2^ = 6.87, *P* = .009) were all more common in the peritoneal metastasis group. Imaging and intraoperative findings showed similar trends. Ascites (68.4% vs 19.8%; χ^2^ = 31.16, *P* < .001), peritoneal thickening or nodules (63.2% vs 12.1%; χ^2^ = 40.20, *P* < .001), and omental caking (39.5% vs 3.4%; χ^2^ = 34.35, *P* < .001) on CT were markedly more frequent in the peritoneal metastasis group. Intraoperatively, peritoneal or omental implants were identified in 84.2% of patients with peritoneal metastasis compared with 7.8% in those without peritoneal metastasis (χ^2^ = 85.64, *P* < .001; Table 2).

**Table 2:** Tumor-related, pathological, imaging, and intraoperative characteristics in patients with and without peritoneal metastasis.

Variable	Peritoneal metastasis group (n = 38)	Non-peritoneal metastasis group (n = 116)	Test statistic	*P*-value
Tumor-related and pathological characteristics				
Tumor size, cm, mean ± SD	6.7 ± 1.8	4.7 ± 1.7	*t* = 5.85	<.001
Tumor location, n (%)				
Proximal (cardia/fundus)	10 (26.3%)	20 (17.2%)	χ^2^ = 1.50	.472
Middle (body)	9 (23.7%)	31 (26.7%)		
Distal (antrum/pylorus)	19 (50.0%)	65 (56.0%)		
T4 invasion (vs T3), n (%)	30 (78.9%)	58 (50.0%)	χ^2^ = 9.79	.002
N2–3 nodal stage (vs N0–1), n (%)	28 (73.7%)	49 (42.2%)	χ^2^ = 11.32	<.001
Lauren classification, n (%)				
Intestinal	10 (26.3%)	62 (53.4%)	χ^2^ = 15.16	<.001
Diffuse	22 (57.9%)	28 (24.1%)		
Mixed/indeterminate	6 (15.8%)	26 (22.4%)		
Poor or signet-ring differentiation, n (%)	30 (78.9%)	63 (54.3%)	χ^2^ = 7.26	.007
Lymphovascular invasion present, n (%)	29 (76.3%)	53 (45.7%)	χ^2^ = 10.78	.001
Perineural invasion present, n (%)	24 (63.2%)	45 (38.8%)	χ^2^ = 6.87	.009
Imaging and intraoperative findings				
Ascites on CT, n (%)	26 (68.4%)	23 (19.8%)	χ^2^ = 31.16	<.001
Peritoneal thickening or nodules on CT, n (%)	24 (63.2%)	14 (12.1%)	χ^2^ = 40.20	<.001
Omental caking on CT, n (%)	15 (39.5%)	4 (3.4%)	χ^2^ = 34.35	<.001
Intraoperative peritoneal or omental implants, n (%)	32 (84.2%)	9 (7.8%)	χ^2^ = 85.64	<.001

T and N refer to tumor and nodal categories of the TNM staging system.

CT = computed tomography, LVI = lymphovascular invasion, PNI = perineural invasion, SD = standard deviation.

### 3.3. Univariate analysis of risk factors for peritoneal metastasis

Univariate logistic regression was performed to explore factors associated with peritoneal metastasis in patients with advanced gastric cancer (Table 3). Age ≥ 60 years and male sex were not significantly associated with peritoneal metastasis (both *P* > .05). In contrast, poorer performance status (ECOG PS ≥ 2), larger tumor size (≥5 cm), T4 invasion, and advanced nodal stage (N2–3) were all significantly associated with an increased odds of peritoneal metastasis. Histopathological characteristics, including diffuse Lauren type, poor or signet-ring cell differentiation, lymphovascular invasion, and perineural invasion, were also significantly related to peritoneal dissemination. Imaging and intraoperative variables showed the strongest associations: the presence of ascites, peritoneal thickening or nodules, omental caking on CT, and intraoperative peritoneal or omental implants markedly increased the odds of peritoneal metastasis, with intraoperative implants showing the highest odds ratio.

**Table 3:** Univariate analysis of factors associated with peritoneal metastasis in patients with advanced gastric cancer.

Variable	OR	95% CI	*P*-value
Age ≥ 60 yr (vs < 60)	0.90	0.43–1.88	.785
Male sex (vs female)	1.14	0.52–2.50	.743
ECOG PS ≥ 2 (vs 0–1)	2.83	1.32–6.08	.007
Tumor size ≥ 5 cm (vs < 5 cm)	5.92	2.49–14.05	<.001
T4 invasion (vs T3)	3.75	1.59–8.87	.002
N2–3 nodal stage (vs N0–1)	3.83	1.70–8.61	<.001
Diffuse Lauren type (vs intestinal/mixed)	4.32	2.00–9.35	<.001
Poor/signet-ring differentiation (vs well/moderate)	3.15	1.33–7.46	.007
LVI present (vs absent)	3.83	1.67–8.80	.001
PNI present (vs absent)	2.70	1.27–5.77	.009
Ascites on CT (vs absent)	8.76	3.85–19.94	<.001
Peritoneal thickening/nodules on CT (vs absent)	12.49	5.26–29.63	<.001
Omental caking on CT (vs absent)	18.26	5.55–60.07	<.001
Intraoperative peritoneal/omental implants (vs absent)	63.41	20.98–191.62	<.001

T and N refer to the tumor and nodal categories of the TNM staging system.

CI = confidence interval, CT = computed tomography, ECOG PS = Eastern Cooperative Oncology Group performance status, LVI = lymphovascular invasion, OR = odds ratio, PNI = perineural invasion.

### 3.4. Multivariate logistic regression analysis

Variables that were statistically significant in univariate analysis or considered clinically relevant (ECOG PS ≥ 2, tumor size ≥ 5 cm, T4 invasion, N2–3 nodal stage, diffuse Lauren type, lymphovascular invasion, and peritoneal thickening or nodules on CT) were entered into a multivariate logistic regression model (Table 4). After adjustment, tumor size ≥ 5 cm, diffuse Lauren type, lymphovascular invasion, and peritoneal thickening or nodules on CT remained independently associated with peritoneal metastasis. Tumor size ≥ 5 cm was associated with approximately a 3.5-fold increased odds of peritoneal metastasis (OR = 3.49, 95% confidence interval [CI]: 1.63–7.50, *P* = .001). Patients with diffuse Lauren type had higher odds of peritoneal dissemination than those with intestinal/mixed type (OR = 3.32, 95% CI: 1.49–7.42, *P* = .003). The presence of lymphovascular invasion was also an independent risk factor (OR = 2.59, 95% CI: 1.23–5.45, *P* = .012). Peritoneal thickening or nodules on CT showed the strongest independent association with peritoneal metastasis (OR = 4.95, 95% CI: 2.17–11.28, *P* < .001). In contrast, ECOG PS ≥ 2, T4 invasion, and N2–3 nodal stage did not retain statistical significance in the multivariate model (all *P* > .05).

**Table 4:** Multivariate logistic regression analysis of factors associated with peritoneal metastasis in patients with advanced gastric cancer.

Variable	β	SE	Wald χ^2^	*P*-value	OR	95% CI for OR
ECOG PS ≥ 2 (vs 0–1)	0.55	0.36	2.33	.127	1.73	0.86–3.51
Tumor size ≥ 5 cm (vs < 5 cm)	1.25	0.39	10.27	.001	3.49	1.63–7.50
T4 invasion (vs T3)	0.40	0.35	1.31	.253	1.49	0.75–2.96
N2–3 nodal stage (vs N0–1)	0.45	0.34	1.75	.186	1.57	0.81–3.05
Diffuse Lauren type (vs intestinal/mixed)	1.20	0.41	8.57	.003	3.32	1.49–7.42
LVI present (vs absent)	0.95	0.38	6.25	.012	2.59	1.23–5.45
Peritoneal thickening/nodules on CT (vs absent)	1.60	0.42	14.51	<.001	4.95	2.17–11.28

T and N refer to the tumor and nodal categories of the TNM staging system.

β = regression coefficient, CI = confidence interval, CT = computed tomography, ECOG PS = Eastern Cooperative Oncology Group performance status, LVI = lymphovascular invasion, OR = odds ratio, SE = standard error.

## 4. Discussion

The present study systematically evaluated clinicopathological and radiological factors associated with peritoneal metastasis in patients with advanced gastric cancer. In multivariate analysis, tumor size ≥ 5 cm, diffuse Lauren type, lymphovascular invasion, and peritoneal thickening or nodules on CT emerged as independent risk factors for peritoneal dissemination, whereas ECOG PS, T4 invasion, and advanced nodal stage lost significance after adjustment. These findings suggest that both intrinsic tumor biology and patterns of local and serosal spread, as reflected by histopathology and CT, jointly determine the propensity for peritoneal metastasis.

The association between larger primary tumors and peritoneal metastasis is biologically plausible. Increased tumor volume is generally accompanied by deeper mural invasion, a higher probability of serosal penetration, and a larger tumor–peritoneal interface, all of which facilitate exfoliation of malignant cells into the peritoneal cavity. The higher proportion of T4 and N2–3 disease in the peritoneal metastasis group in univariate analyses is consistent with this concept. However, once tumor size and other factors were entered into the multivariate model, T4 stage and nodal status were no longer independent predictors, which suggests that their effect may be mediated through tumor burden and associated vascular and lymphatic invasion rather than acting as isolated determinants. Histopathological characteristics in this cohort further support the central role of aggressive tumor biology in peritoneal spread. The diffuse Lauren subtype, poor or signet-ring cell differentiation, and the presence of lymphovascular and perineural invasion were all more frequent in patients with peritoneal metastasis. Diffuse-type tumors, which are characterized by poorly cohesive cells and frequent signet-ring morphology, tend to infiltrate the gastric wall extensively, producing early serosal involvement and microscopic peritoneal implantation. Lymphovascular invasion represents a marker of high invasive potential and systemic dissemination, and its independent association with peritoneal metastasis in the multivariate model indicates that it may also reflect a tumor phenotype prone to trans-serosal spread and implantation within the peritoneal cavity. Perineural invasion, although not an independent factor in the adjusted model, showed a significant univariate association and may be a surrogate for an aggressive growth pattern.

Radiologic and intraoperative indicators showed strong associations with peritoneal dissemination. Ascites, peritoneal thickening or nodules, and omental caking on CT were markedly more frequent in the peritoneal metastasis group, and peritoneal thickening or nodules remained an independent predictor after adjustment. These findings indicate that even within a population of advanced gastric cancer, macroscopic and microscopic peritoneal involvement manifests in recognizable CT patterns that can be integrated into risk stratification. Intraoperative detection of peritoneal or omental implants was strongly concordant with radiologic signs, reinforcing the diagnostic relevance of careful preoperative CT review for peritoneal features. Importantly, the fact that CT-based peritoneal thickening or nodularity retained significance in multivariate analysis suggests that radiologic assessment adds information beyond traditional pathological variables and may capture subclinical or early peritoneal involvement that is not fully reflected by T and N stage alone. The lack of an independent effect of ECOG PS, T4, and N2–3 stage in the multivariate model likely reflects confounding and collinearity. Patients with poor performance status or advanced T/N stage frequently have larger, more infiltrative tumors with lymphovascular invasion and radiologic evidence of peritoneal involvement. Once these more proximal determinants of metastatic behavior are accounted for, ECOG PS and anatomic stage may no longer contribute additional discriminatory value for peritoneal metastasis per se, even though they remain important prognostic variables for overall survival and treatment tolerance.

Several recent investigations have addressed risk factors for peritoneal carcinomatosis in gastric cancer, and the present results are broadly consistent with these data. A recent systematic review and meta-analysis of staging laparoscopy in gastric cancer identified T4 stage, N2/3 stage, poor differentiation, large tumor size, and Lauren diffuse-type as significant risk factors for peritoneal carcinomatosis, with large tumor diameter and diffuse histology yielding relatively high pooled odds ratios.^[10]^ Our finding that tumor size ≥ 5 cm and diffuse Lauren type are independent predictors aligns closely with these pooled estimates and supports the use of these features to select candidates for staging laparoscopy. Population-based and institutional studies published in the last few years have similarly highlighted advanced T stage, nodal involvement, and high-grade or poorly cohesive histology as determinants of synchronous or metachronous peritoneal metastasis in gastric cancer.^[1,11]^ In particular, diffuse and mixed Lauren subtypes have been associated with higher risks of peritoneal recurrence and poorer survival compared with the intestinal subtype, even after controlling for stage and treatment modality.^[12]^ The strong association between diffuse-type and peritoneal metastasis in our multivariable analysis is concordant with these observations and reinforces the concept that Lauren classification captures clinically meaningful biological heterogeneity relevant to patterns of spread.

The independent role of lymphovascular invasion in the current study is also in line with recent evidence. Contemporary series have reported lymphovascular invasion as an important prognostic indicator and an independent predictor of recurrence in resected gastric cancer, including peritoneal relapse and distant metastasis.^[13]^ Our data extend this evidence by showing that lymphovascular invasion is not only associated with worse overall prognosis but is specifically linked to peritoneal dissemination in the setting of advanced disease. Regarding imaging, several recent studies have focused on CT-based assessment and radiomics for prediction of occult peritoneal metastasis. Conventional CT signs such as ascites, peritoneal thickening or nodularity, and omental caking are repeatedly reported as highly specific, although not uniformly sensitive, markers of peritoneal involvement in gastric cancer.^[8]^ More recent radiomics and machine learning approaches have incorporated texture and semantic features of the primary tumor and peritoneum to improve detection of occult peritoneal metastasis beyond these direct signs.^[8,14]^ The present study, although based on conventional visual assessment rather than radiomics, confirms that peritoneal thickening or nodularity on CT carries a strong independent association with peritoneal metastasis. This is consistent with emerging evidence that integrating CT-derived peritoneal features with clinicopathological variables can substantially improve preoperative risk stratification for peritoneal dissemination.

Recent reviews on the biology and management of gastric cancer with peritoneal metastasis emphasize that peritoneal dissemination reflects complex interactions between tumor cell adhesion, epithelial–mesenchymal transition, stromal remodeling, and the peritoneal microenvironment.^[15,16]^ Our observation that diffuse-type histology and lymphovascular invasion are associated with peritoneal spread is compatible with these mechanistic insights because these phenotypes are often associated with enhanced migratory capacity, loss of cell–cell adhesion, and a permissive stromal milieu for peritoneal implantation. Overall, the present findings are concordant with contemporary literature and add further quantitative evidence from a well-characterized cohort, particularly with respect to combining histopathological and CT features in a single multivariable framework.

This study has several strengths. First, it focuses specifically on peritoneal metastasis in a homogeneous cohort of advanced gastric cancer patients managed at a single institution, which reduces variability in diagnostic and treatment pathways. Second, the analysis integrates clinical, histopathological, imaging, and intraoperative variables within a multivariate logistic regression framework, allowing identification of independent predictors while accounting for confounding between tumor burden, biological aggressiveness, and radiologic findings. Third, the study uses routinely available parameters – tumor size, Lauren type, lymphovascular invasion, and standard CT features – which facilitates translation of the results into everyday clinical practice without the need for specialized assays or technology. However, several limitations should be acknowledged. The retrospective, single-center design introduces inherent risks of selection bias and residual confounding, and causal inferences cannot be established. The sample size, although sufficient to detect several independent associations, may limit the precision of estimates for less common variables and preclude detailed subgroup analyses by treatment modality or molecular subtype. Radiologic assessments were based on conventional CT interpretations and did not incorporate radiomics or advanced imaging modalities, which may provide additional predictive information in future studies. Furthermore, molecular and genomic markers that may influence peritoneal tropism were not evaluated, and external validation in independent cohorts is needed to confirm the generalizability of the identified risk factors across different populations and practice settings. Because the diagnosis of peritoneal metastasis was based on multiple clinical modalities (CT findings, staging laparoscopy, cytology, or intraoperative assessment) rather than a single uniform reference standard, misclassification bias may have occurred. In particular, patients evaluated primarily by imaging may have had occult microscopic peritoneal disease that was not detected, potentially resulting in underestimation of peritoneal metastasis in some cases, while certain radiologic findings may also have lacked absolute specificity. This heterogeneity in diagnostic confirmation could have introduced nondifferential classification error and influenced effect estimates. Future prospective studies incorporating standardized staging laparoscopy and cytological assessment are warranted to improve diagnostic consistency.

Although several significant predictors were identified, the number of peritoneal metastasis events (38 cases) relative to the number of variables entered into the multivariate logistic regression model may have resulted in a limited events-per-variable ratio. This raises the possibility of model overfitting, unstable regression coefficients, and reduced generalizability. Therefore, the identified associations should be interpreted with caution, and validation in larger multicenter cohorts with more robust event numbers is necessary.

## 5. Conclusion

In conclusion, this study demonstrates that larger tumor size, diffuse Lauren type, lymphovascular invasion, and CT-detected peritoneal thickening or nodules are independent risk factors for peritoneal metastasis in advanced gastric cancer. Incorporating these readily obtainable clinicopathological and imaging features into preoperative assessment may improve risk stratification and assist in selecting patients for staging laparoscopy and individualized treatment strategies.

## Author contributions

**Conceptualization:** Airu Su, Ran Guo, Yuhan Meng, Ye Li, Chao Wu.

**Data curation:** Airu Su, Ran Guo, Yuhan Meng, Ye Li, Chao Wu.

**Formal analysis:** Airu Su, Ran Guo, Yuhan Meng, Ye Li, Chao Wu.

**Funding acquisition:** Airu Su, Chao Wu.

**Writing – original draft:** Airu Su, Chao Wu.

**Writing – review & editing:** Airu Su, Chao Wu.
